# Factors important in the choice of a medical career: a Finnish national study

**DOI:** 10.1186/s12909-015-0451-x

**Published:** 2015-10-05

**Authors:** Teppo J. Heikkilä, Harri Hyppölä, Jukka Vänskä, Tiina Aine, Hannu Halila, Santero Kujala, Irma Virjo, Markku Sumanen, Kari Mattila

**Affiliations:** 1 Unit of Primary Health Care, Hospital District of Northern Savo, P.O. Box 100, Kuopio, FI 70029 Finland; 2 Emergency Department, Kuopio University Hospital, P.O. Box 100, Kuopio, FI 70029 Finland; 3 Finnish Medical Association, P.O. Box 49, Helsinki, FI 00501 Finland; 4 Department of General Practice, School of Medicine, University of Tampere, Kalevantie 4, Tampere, FI 33014 Finland; 5 Centre of General Practice, Pirkanmaa Hospital District, P.O. Box 2000, Tampere, FI 33521 Finland

## Abstract

**Background:**

Applying for medical school is the first and also one of the most important career choices a physician makes. It is important to understand the reasons behind this decision if we are to choose the best applicants for medical schools and enable them to pursue satisfying careers.

**Methods:**

Respondents to the Finnish Junior Physician 88, Physician 1998 and Physician 2008 studies were asked: “*To what extent did the following factors influence your decision to apply for medical school?*” In 1998 and 2008 the respondents were also asked: “*If you were starting your studies now, would you start studying medicine?*” and had to answer “*Yes*” or “*No*”. The odds ratios for the answer “*No*” were tested using logistic regression models.

**Results:**

*"Interest in people*” was the main motive for starting to study medicine. “*Good salary*” and “*Prestigious profession*” were more important motives for males and “*Vocation*” and “*Interest in people*” for females. There were some significant changes in the motives for entering medicine in the 20-year period between studies. “*Vocation*” and “*Wide range of professional opportunities*” as important motives for entering medicine predicted satisfaction with the medical profession.

**Discussion:**

Strong inner motivation may indicate the ability to adapt to the demands of work as a physician.

**Conclusions:**

Medical schools should try to select those applicants with the greatest vocational inclination towards a medical career.

## Background

Deciding to apply for medical school is the first but also one of the most important career choices that a physician will ever make. It is important to understand the reasons behind this choice, and also the consequences.

The decision to be a physician is often made in the early stages of life [1]. For example, it has been found that a quarter of clinicians had decided that they would be applying for medical school even before attending high school [2].

There are more candidates applying to study medicine than there are places available, and this means that the selection procedure needs to be relevant, reliable and fair [3]. However, there may be wide variations in the different application and selection procedures even in one country’s universities [4]. It is important that applicants seeking a place at medical school should do so for the right reasons [5]. This is because selectors have to assess applicants’ ability to acquire clinical skills and assume the professional attitude appropriate for practice [6]. There is a need to assess medical school applicants for their ability to become the kind of physicians people want. The motives for choosing a career in medicine also seem to remain relatively stable during medical school [7]. Furthermore, there has been some debate about the usefulness and predictivity of aptitude tests or personal interviews, and even about personality testing during the process of selecting students for medical school [4, 6, 8–14]. It has been shown that there are large variations in personality among medical students, and these differences also affect the students’ performance during studies, even if medical students overall seem to be more social and empathic than other students [15]. In addition, dissatisfaction in practising medicine is a significant predictor of how physicians perceive their professional responsibilities and in medical decision-making, and also has significant implications for the quality of care [16–18].

There seem to be some differences between genders in terms of the motives for choosing to apply for medical school [19–21]. This is not surprising, since there are also differences in personal values between male and female physicians [22]. In Finland, just over half (56 %) of all applicants were female in 2013, although the proportion of females has declined somewhat in recent years [23, 24]. In any case, since more females are entering medicine, these differences need to be addressed.

In Finland, applying for medical school is arranged via an entrance exam held once a year. The exam is the same for all five medical schools, and an applicant may apply to only one medical school at a time. Some of the performance in the national final examinations from secondary school is also taken into account in the selection of medical students. Aptitude tests are not used. In recent years, the number of applicants has been increasing [23, 24]. In 2013, only about one out of every seven applicants (15 %) was accepted into medical school [24]. In Finland, approximately 95 % of those accepted into medical school also graduate.

It is important to identify and recognise the motives why physicians actually apply for medical school and what are the consequences of the different motives, since this would reveal whether the selection criteria are choosing the best applicants. This kind of information is necessary if we are to develop the application procedures for medical schools to meet the increasing demands placed on physicians working in 21st century health care.

The aim of this study was to determine the main motives behind the choice of medicine as a career by Finnish physicians and to identify the reasons for dissatisfaction with the chosen profession. Another goal was to compare the responses in 1988, 1998 and 2008 to see if there were any changes in these motives over the past twenty years.

## Methods

The Junior Physician 88, Physician 1998 and Physician 2008 studies were undertaken in collaboration with the University of Kuopio (now the University of Eastern Finland), the University of Tampere and the Finnish Medical Association. The cross-sectional studies compiled information on the social background, work history, employment and career plans of the medical profession in Finland. The studies also examined physicians’ views of basic and postgraduate education, their values and professional identity. The questions were mostly formed before the first study in 1988 by the study group, although some new questions were added in later questionnaires. Most of the questions have been in the same format since then to ensure comparability between studies.

In all Physician 1988, 1998, and 2008 studies, the basic study population consisted of all medical doctors licensed in Finland 2–11 years before the study. Each study covered a random sample of physicians based on their date of birth. Addresses were collected from the Finnish Medical Association’s database, which covers all physicians licensed in Finland. The basic characteristics of the data from the 1988, 1998, and 2008 studies are presented in Table 1. The basic reports of the studies have been published by the Finnish Ministry of Social Affairs and Health [25–27]. In the 1988 and 1998 studies, the data well represented physicians licensed in Finland in terms of age and gender [26, 27]. In the 2008 study more women answered the questionnaire than men, and the response rate also increased slightly with age [25]. For these reasons, the 2008 data was weighted by age and gender.

**Table 1:** Data for the young physician 88, physician 1998 and physician 2008 studies

	1988	1998	2008
Study population	5,208	4,926	5,092
Study sample	2,632	2,492	2,401
Returned questionnaires	1,745	1,822	1,211
Response rate (%)	66.3	73.1	50.4

Data for the 1988 and 1998 studies was collected by postal questionnaire. In the 2008 study both postal and online questionnaires were used [28]. Of the responses, 46 % were submitted online and 54 % via the postal questionnaire. Responses to the questionnaire were anonymous, and all answers were treated confidentially. According to Finnish legislation, studies of this kind do not need ethical approval, since they do not affect the respondent’s personal integrity and as respondents are free to choose whether to respond or not [29, 30]. Respondents were fully informed about the use of the questionnaire in the cover letter. Therefore, it was presumed that respondents gave an informed consent when choosing to answer the questionnaire.

Respondents were asked: “*To what extent did the following factors influence your decision to apply for a medical school?*” and were offered a total of eleven items which could have affected their choice. The data were obtained by means of a Likert five-point scale (*not at all*, *slightly*, *to some extent*, *quite a lot*, *very much*). For this study, *quite a lot* and *very much* were defined as “important” motives (*a lot*), and *not at all* and *slightly* as “not important” motives (*hardly at all*). In the 2008 and 1998 studies the respondents were also asked: “*If you were starting your studies now, would you start studying medicine?*” and had to answer “*Yes*” or “*No*”.

We compared the motives behind the choice of medicine between respondents in 1988, 1998, and 2008. We then compared the answers and the differences between genders in 1988, 1998, and 2008. We also looked to see if there were any differences between those who would start studying medicine again, and those who would not, in terms of their motives to start studying medicine in 1998 and 2008. The significances of differences were tested using the *chi-squared* test.

Finally, the odds ratios for the answer “*No*” to the question: “*If you were starting your studies now, would you start studying medicine?*” in the 1998 and 2008 studies were tested using logistic regression models. The models included the independent variables gender, age, and time elapsing since being licensed as a physician, as well as the six most frequent motives for applying for medical school (*Interest in people, Prestigious profession, Wide range of professional opportunities, Vocation, Good salary,* and *Achievements at school*). *Nagelkerke’s R-squared* and the *Hosmer-Lewenshow* tests were calculated for both logistic regression models. The data were analysed using SPSS Statistics 19.0.0.1 for Macintosh predictive analytics software.

## Results

**Fig. 1:**
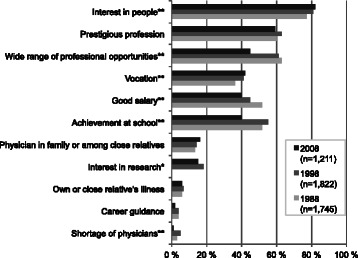
Motives for applying for medical school in 1988, 1998, and 2008 studies. Proportions (%) of respondents answering “*Quite a lot*” or “*Very much*” to the question “*To what extend did the following motives influence your decision to apply for medical school?*” in Young Physician 88, Physician 1998, and Physician 2008 studies. *Note* **p < 0.01 and *p < 0.05 for the difference between study years. “*Interest in research*” was not asked in the Young Physician 88 study

*“Interest in people”* was the main motive for wanting to study medicine in all 1988, 1998 and 2008 studies. This was an important motive for 77–82 % of respondents in choosing medicine as a career (Fig. 1). The importance of “*Prestigious profession”* was also high in all three studies with three out of five respondents claiming this as an important motive. The relative importance of “*Vocation”* increased (from 36 % to 42 %) between 1988 and 2008. At the same time there were declines in “*Wide range of professional opportunities”* (from 63 % to 45 %), “*Good salary”* (from 52 % to 40 %), and “*Achievements at school*” (from 52 % to 40 %).

There were considerable differences between males and females in terms of their response to the question about their motives (Table 2). “*Good salary”* and “*Prestigious profession”* were significantly more important motives for males, whereas “*Vocation”*, “*Interest in people”*, “*Achievements at school”*, and “*Career guidance”* were significantly more important for females in all three studies. “*A physician in family or among close relatives*” was significantly more important for males, and “*Career guidance*” for females in 1998 and 2008 studies.

**Table 2:** Motives of males and females for applying for medical school

	Males				Females				Differences between genders		
	1988	1998	2008	Sig.	1988	1998	2008	Sig.	1988	1998	2008
n	889-892	577-582	322-325		835-839	1,222-1,226	874-880				
	%	%	%	*p*	%	%	%	*p*	%-units	%-units	%-units
Good salary	56	49	49	**	47	43	36	**	9**	6*	13**
Prestigious profession	64	66	65		56	61	56	*	8**	5*	9**
A physician in family or among close relatives	15	18	21	*	11	13	14		4	5**	7**
Interest in research		19	17			17	14			2	3
Shortage of physicians	4	6	2	**	4	5	1	**	0	1	1
Wide range of professional opportunities	61	58	43	**	64	63	45	**	−3	−5*	−2
Own or close relative’s illness	6	5	4		7	8	6		−1	−3**	−2
Career guidance	3	2	1	*	4	4	3		−1	−2*	−2**
Achievements at school	47	46	35	**	58	60	42	**	−11**	−14**	−7*
Interest in people	71	72	74		83	85	84		−12**	−13**	−10**
Vocation	30	32	35		42	45	45		−12**	−13**	−10**

Proportions of males and females who answered “*Quite a lot*” or “*Very Much*” to the question “*To what extent did the following factors influence your desicion to apply for a medical school?*”, and differences in propotions in Junior Physician 88, Physician 1998, and Physician 2008 studies. The items are sorted by the difference between genders in 2008. *Note* **p < 0.01, *p < 0.05. “*Interest in research”* was not asked in Junior Physician 88 study

In the Physician 2008 study 17 % of respondents would not have started studying medicine again if making the decision now. This proportion had decreased from 25 % in the 1998 study. The question was not asked in the 1988 study. Subdivided by gender, 13 % of male respondents would not have started studying medicine again in 2008, compared with 26 % in 1998. For female physicians, the corresponding figures were 18 % in 2008 and 25 % in 1998. All changes were statistically significant (*p* < 0.01).

“*Vocation*”*,* “*Wide range of professional opportunities*”, and “*Interest in people*” were significantly more important reasons for applying for medical school for those who would still choose medicine if they were beginning their studies now, compared to those who would not in both 1998 and 2008 studies (Table 3).

**Table 3:** Motives of Yes- and No-groups for applying for medical school

	YES (Would start studying medicine again)			NO (Would not start studying medicine again)			Differences between groups	
	1998	2008	Sig.	1998	2008	Sig.	1998	2008
n	1,281-1,286	980-987		437-441	196-198			
	%	%	*p*	%	%	*p*	%-units	%-units
Vocation	47	45		27	26		20**	19**
Wide range of professional opportunities	64	47	**	53	31	**	11**	16**
Interest in people	84	83		73	71		11**	12**
Prestigious profession	64	60	*	59	58		5	2
A physician in family or among close relatives	15	17		13	14		2	3
Good salary	44	41		48	41	*	−4	0
Own or close relative’s illness	6	6		8	6		−2	0
Career guidance	3	2		5	2		−2	0
Shortage of physicians	4	1	**	9	3	**	−5**	−2*
Achievements at school	56	39	**	56	44	**	0	−5
Interest in research	17	14	*	17	21		0	−7*

Motives for applying for medical school (answers “*Quite a lot*” and “*Very much*” to the question “*To what extent did the following factors influence your desicion to apply for a medical school?*”) of those who answered “*Yes*” or “*No*” to the question “*If you were starting your studies now, would you start studying medicine?*” in the Physician 1998 and Physician 2008 studies. The items are sorted by the difference in 2008. *Note* **p < 0.01, *p < 0.05. The question was not asked in the Junior Physician 1988 study

In the 2008 study female physicians had significantly higher odds ratio to answer “*No”* to the question “*If you were starting your studies now, would you start studying medicine?”* (Table 4). In the 1998 study the odds ratio for the 30–34 year old respondents was significantly higher than for those under 30. In the 2008 study there were no significant differences between age groups. The time elapsed since being licensed as a physician had no significance.

When we compared respondents with different motives for applying for medical school, we found those who reported that the motives “*Wide range of professional opportunities*” and “*Vocation*” greatly affected their choice had a significantly lower odds ratio for the answer “*No*” in both studies. In 1998, those for whom the motive “*Interest in people*” greatly affected their desicion to apply for a medical school had a significantly lower odds ratio for the answer “*No*” to the question “*If you were starting your studies now, would you start studying medicine?”* compared to those who answered “*Hardly at all*”. Respectively, in 2008 the odds ratio was significantly lower compared to those who answered “*To some extent*”. In the 1998 study, those who thought that “*Interest in people*” had had little effect on their decision to start studying medicine the odds ratio for the answer “*No*” to the question “*If you were starting your studies now, would you start studying medicine?”* was significantly higher compared to those who thought “*Interest in people*” had greatly affected their decision. On the other hand, in the 1998 study for those respondents who thought “*Good salary*” had had hardly any effect on their decision to start studying medicine the odds ratio for the answer “*No*” was significantly lower compared to those respondents who reported “*Good salary*” to have had a major effect.

**Table 4:** The odds ratios for not starting studying medicine if making the decision now

			1998		2008	
			n	OR (95 % C.I.)	n	OR (95 % C.I.)
*Gender*		Males	560	1	318	1
		Females	1,156	1.16 (0.90 to 1.49)	857	**1.92 (1.33 to 2.77)**
*Age*		Under 30	260	1	167	1
		30-34	776	**1.62 (1.13 to 2.32)**	490	1.25 (0.71 to 2.20)
		35-39	573	1.14 (0.74 to 1.78)	406	1.25 (0.64 to 2.44)
		40 or older	107	1.28 (0.69 to 1.22)	112	1.18 (0.76 to 1.81)
*Time elapsed from the qualification as a physician*		2-6 years	908	1	589	1
		7-11 years	808	0.92 (0.69 to 1.22)	586	1.17 (0.76 to 1.81)
*The most frequent motives for applying for medical school:*						
	*Interest in people*	A lot	1,386	1	955	1
		To some extent	209	1.22 (0.88 to 1.71)	154	**1.57 (1.03 to 2.40)**
		Hardly at all	121	**1.91 (1.27 to 2.88)**	66	1.53 (0.85 to 2.78)
	*Prestigious profession*	A lot	1,079	1	693	1
		To some extent	424	1.21 (0.88 to 1.67)	339	1.00 (0.66 to 1.52)
		Hardly at all	213	**1.76 (1.13 to 2.75)**	143	1.29 (0.71 to 2.32)
	*Wide range of professional opportunities*	A lot	1,049	1	524	1
		To some extent	310	**1.51 (1.12 to 2.02)**	309	**1.70 (1.13 to 2.55)**
		Hardly at all	357	**1.46 (1.10 to 1.95)**	342	**2.44 (1.67 to 3.57)**
	*Vocation*	A lot	708	1	495	1
		To some extent	369	**1.45 (1.05 to 2.00)**	275	**1.84 (1.19 to 2.84)**
		Hardly at all	639	**2.57 (1.95 to 3.38)**	405	**2.64 (1.77 to 3.93)**
	*Good salary*	A lot	764	1	464	1
		To some extent	552	0.75 (0.56 to 1.02)	451	0.88 (0.59 to 1.32)
		Hardly at all	400	**0.59 (0.40 to 0.86)**	260	0.76 (0.45 to 1.28)
	*Achievements at school*	A lot	952	1	475	1
		To some extent	357	0.79 (0.58 to 1.08)	326	1.00 (0.66 to 1.50)
		Hardly at all	407	1.00 (0.74 to 1.35)	374	0.99 (0.66 to 1.49)
*Nagelkerke’s R-squared*				0.103		0.098
*Hosmer-Lemeshow*				0.138		0.923

The odds ratios with 95 % confidence intervals in binary logistic regression model for answering “*No*” to the question: “*If you were starting your studies now, would you choose to be a physician?*” in Physician 1998 and Physician 2008 studies. *Note* “statistically” significant (*p* < 0.05) values are in BOLD. The question was not asked in Junior Physician 88 study

## Discussion

“*Interest in people*” was the main reason for choosing a career in medicine in all three studies. “*Vocation*”, and “*Wide range of professional opportunities*” predicted that respondents would still have chosen medicine as a profession, although these were not the most important overall motives. The motives for applying for medical school showed some significant changes during the 20-year period between studies.

The predicted subsequent satisfaction related to “*Vocation*” and “*Wide range of professional opportunities*” may indicate that those who have a strong inner motivation to work in the medical profession are also able to adapt to the sometimes challenging conditions faced by physicians. Conversely, those who viewed these motives as less important might more often have preferred to embark on a different career. Selecting students who are more likely to be satisfied with medicine as a career would most probably also lead to longer careers and better quality of care [31, 32]. However, addressing these motives in the student selection might not be an easy task since we are not aware of any selection tools that are reliable in screening for them. Still, this adds a new perspective into the discussion about the need to develope the selection processes of medical schools.

The importance of “*Interest in people*” as the main motive for applying for medical school has been the same for twenty years, despite the evident changes between generations [33, 34]. It has also been found that motives related to people and work content are important for physicians [19, 35, 36]. The opportunity to help others has also been the most influential factor for choosing medicine cited by medical students elsewhere [20]. These findings indicate that the most important motive for choosing a medical career is still the content of the work and the profession itself. It has also been stated that in order to be a successful physician one needs to be person-centred and to have a comprehensive and holistic approach [37, 38]. Despite all the evident changes taking place in health care and the society, this still represents a solid basis for undertaking medical education and subsequent success in this profession.

The proportion of respondents who would not apply for medical school if they were making the choice again had decreased significantly between the 1998 and 2008 studies, especially among male respondents. However, in the 2008 study, females had a significantly greater odds ratio than males to be unhappy with their chosen profession. The reasons for these findings are unclear, but may originate from changes in working life. The main difference in physicians’ working life in Finland between these study years was that especially in the early 1990s there was unemployment while in the 2000s there has been a shortage of physicians. This means that in the 2000s physicians had much more opportunities to choose where and how they wanted to work [39]. This may to some extent explain the increase in satisfaction, but it is hard to deduce that it is also related to the greater odds ratio of not applying for medical school again of female physicians. In any case, this is an important question that requires further study, especially as more women are entering medicine.

There were some significant changes between these three studies arranged over a period of twenty years with respect to physicians’ motives affecting the decision to start studying medicine. The relative importance of “*Good Salary*” and “*Achievements at school*” had decreased as motives for entering medicine. Since the importance of “*Vocation*” had increased at the same time, it seems likely that other factors, such as salary and other people’s expectations, are less important inducements for the younger generation of physicians. At the same time, the relative importance of “*Wide range of professional opportunities*” has also decreased suggesting for example that the younger physicians may be more oriented to a particular medical career when entering the medical school.

The strength of this study is that it provides national data from three time periods over 20 years. However, there are obviously some limitations. First of all, when the first study was conducted in 1988, there were few other studies addressing this issue or requirements to validate the questionnaire. Since then the questionnaires have been largely the same in order to aid comparability. With questionnaires of this kind, one needs to acknowledge the possible bias stemming from the respondents’ self-reporting. Respondents may in some cases complete the questionnaire differently when they know the results are going to be seen. For example, some reasons for entering medical school might be more socially acceptable than others, and for this reason some respondents might give more acceptable answers than others.

Answering “*No*” to the question about applying for medical school if making the choice now does not indicate whether the respondent intends to leave the medical profession. No assumptions can therefore be made on this question. Instead, our interpretation is that it indicates dissatisfaction with medicine as a career. Yet, this does not take into account the reasons behind the possible dissatisfaction. For example, changes in health status or family circumstances may have made some physicians wish they had chosen a different career, even if they still find their work interesting as such. The terms used in the three studies were not explained in the questionnaires. Therefore we cannot be absolutely sure how the respondents understood the meaning of, for example, “*Wide range of professional opportunities*” as a reason to apply for medical school. From the respondents’ point of view, it might refer to the wide range of different career opportunities within the medical profession, but also to the diverse content of the work of physicians. “*Vocation*” may also be understood in different ways. In this study it is intended to mean an inclination and dedication to follow a career as a physician. Nevertheless, this should not have any major impact on the conclusions of this study.

The response rate was lower in the 2008 study than in the previous studies. However, it is still reasonably comparable to other similar studies [40, 41]. It also needs to be noted that these studies do not offer any longitudinal data. Therefore, comparisons between the different study years need to be conducted with caution.

The values of *Nagelkerke R2* tests for the logistic regression models were rather low. However, the main objective of the logistic regression models used in this study was to estimate the contribution of the independent variables presented. In this respect the models worked well.

In all the Junior Physician 88, Physician 1998, and Physician 2008 studies, the study population covered those physicians who were licensed 2 – 11 years before the study. This means that they had to think back approximately 13 years to the time when they were deciding to apply for medical school and try to remember their reasons at that time. However, it has been reported that important life events remain fairly well fixed in the memory [42]. Since the choice of professional career can be considered such an event, one can assume that tems related to it are well recalled.

## Conclusions

The findings presented here suggest that the most important motives for entering medicine are related to the content of the work as a physician and to medical profession in general. These motives also seem to predict that the subject will have a satisfying career as a physician. In particular, the finding that those applicants citing vocation as a motive are more satisfied with the medical profession should become one of the key factors in the student selection process. Even if it might not be an easy task in practice.
